# A Practical Treatise on the Causes, Symptoms, and Treatment of Spermatorrhea

**Published:** 1858-12

**Authors:** 


ART. VI.—*A Practical Treatise on the Causes, Symptoms, and Treatment of Spermatorrhœa.* By M. LALLEMAND, formerly Professor of Clinical Surgery at the University of Montpellier, etc. Translated and Edited by HENRY J. McDOUGAL, Member of the Royal College of Surgeons of England, etc. Third American Edition; to which is added, *On Diseases of the Vesiculæ Seminales*, and their Associated Organs; with Special Reference to the Morbid Secretions of the Prostrate and Urethral Mucous Membrane. By MARRIS WILSON, M. D. Philadelphia: BLANCHARD & LEA. 1858.

The authors of these works, which have been published by Blanchard & Lea, in one volume, have both truly said in their prefaces, that the subject of which they treat is one which often inspires nothing but disgust in the mind of the practitioner. Spermatorrhœa is certainly not a very inviting field of investigation; but the researches of Lallemand, in this disease, and he is almost the only investigator who has devoted much attention to it, have certainly done much towards alleviating the sufferings, of a character not to be described, of many unfortunate victims of the loathesome vice of masturbation.

The work of Lallemand has passed to its third edition in this country, which, for a work of its class, is an indication of excellent success. Of this it is not necessary to speak in detail; every one is acquainted with the mode of treatment employed by this author, and with the instrument for applying the solid nitrate of silver to the urethra, which bears his name. Its merits have long since been acknowledged by the profession. In reading the editor's preface, we notice that he remarks that the author has not mentioned cases of epilepsy resulting from onanism; this, the editor says, has occurred to him repeatedly. It is certain that nothing is more common than the production of this disease by solitary indulgence; in this number of the Journal we have one of the cases of epilepsy reported by Dr. Hamilton, arising from this cause; we, with the editor, are at a loss to understand this omission on the part of the author.

The work on this subject, by Marris Wilson, which is issued with the work of Lallemand, is much more brief. This has been well received in England, but has never before been published in this country. From the slight examination we have made of it, we have been able to discover nothing very striking excepting a case in which the most heroic treatment was employed for a persistent spermatorrhœa. Here the patient had one testicle extirpated by Sir Astley Cooper; and afterwards the other by another surgeon. Strange to say, these desperate measures were of no avail, and relie was only experienced by the establishment of an immense slough in the perinæum. This reminds us of a case which was treated in this State, and was related to the former editor of this Journal by the attending physician himself. After trying every other means, in an obstinate case of spermatorrhœa, the doctor finally castrated his patient, and *cured him.* We had thought that nothing could be more wonderful than that a patient should be castrated for spermatorrhœa; but the case related by Dr. Wilson, where the patient was castrated and still not cured, is decidedly more remarkable.

